# Who should be included in first-in-human trials? A systematic review of reasons

**DOI:** 10.1186/s12967-025-06550-y

**Published:** 2025-06-11

**Authors:** Lieke van Kempen, Martine C. de Vries, Eelco J. P. de Koning, Nienke de Graeff

**Affiliations:** 1 Department of Medical Ethics and Health Law, Leiden University Medical Center, Leiden, The Netherlands; 2 Department of Internal Medicine, Leiden University Medical Center, Leiden, The Netherlands; 3 LUMC Transplant Center, Leiden University Medical Center, Leiden, The Netherlands

**Keywords:** First-in-human, Clinical trials, Translational medicine, Participant selection, Ethics, Systematic review of reasons, Bench-to-bedside

## Abstract

**Background:**

First-in-human trials mark a significant turning point in translational research, with novel therapies being tested in humans for the first time. Who should be included in FIH trials is a topic of ongoing debate amongst researchers, clinicians, ethicists, and sponsors. Yet, no comprehensive overview of the literature on this topic has been constructed before.

**Methods:**

A systematic review of reasons was conducted, following the methodology outlined by Stretch and Sofaer for conducting systematic reviews of argument-based literature. Six online databases were consulted across various disciplines, including medicine (PubMed and Embase), philosophy (The Philosopher’s Index and PhilPapers), and multidisciplinary studies (Web of Science and Academic Search Premier). Additionally, relevant books and book chapters were identified through the library of Leiden University. After data extraction and analysis, 80 publications were included.

**Results:**

181 reasons were identified amongst six potential participant categories: healthy volunteers, patients (general), patients with less advanced-stage diseases, patients with more advanced-stage diseases, vulnerable populations, and diverse participant groups. These reasons relate to six themes: non-maleficence, beneficence, scientific value, efficiency, respect for persons, and justice. This review highlights multiple challenges in the existing literature, including ambiguous or poorly defined reasons and unspecified use of moral theory, framework, or method, the use of beneficence as an important theme for including participants in FIH trials, and the complexity of identifying and defining vulnerable populations.

**Conclusions:**

This review provides the first comprehensive overview of the reasons for and against including potential participant groups in FIH trials. The results highlight considerations that are relevant to reflect upon when determining and justifying participant selection for FIH trials. Additionally, this review offers guidance for further normative inquiry.

**Supplementary Information:**

The online version contains supplementary material available at 10.1186/s12967-025-06550-y.

## Background

A significant turning point within translational research, where fundamental scientific discoveries are translated into clinical practice, is the transition from pre-clinical to clinical research. First-in-human (FIH) trials represent the first phase of clinical research, with novel drugs or therapeutic interventions being tested in humans for the first time to assess their safety and tolerability [1]. The transition from pre-clinical to clinical research brings new challenges for researchers, clinicians, sponsors, and ethicists. A pivotal moment in this context was the death of 18-year-old Jesse Gelsinger during a FIH gene therapy trial in 1999. His death has shaped ongoing ethical reflection on the protection of research participants in high-risk clinical trials and intensified discussions about the ethical considerations in FIH trials [2]. One intricate ethical challenge related to FIH trials concerns the selection of participants and establishing in- and exclusion criteria. More specifically, sponsors and research teams will have to decide for which participant group(s) participation in the FIH trial can be justified and for what reasons.

To address this challenge, numerous studies have explored the ethics of selecting participants for FIH trials [3–6]. These studies consistently emphasize two crucial factors in participant selection: the balance between risks and benefits and the scientific value of the research. There is broad agreement that ethical participant selection entails including individuals with the most favourable balance between risks and benefits, capable of providing valuable scientific insights. However, determining which participant group(s) meet this (double) criterion remains challenging and can vary across fields and among trials. Furthermore, authors also offer reasons based on other ethical values or criteria that must be considered, such as reasons related to promoting diversity based on the value of justice. This review aims to provide a comprehensive overview of all the reasons for and against including different participant groups in FIH trials by utilizing the existing literature on the ethics of participant selection for FIH trials.^1^

The results of such a comprehensive overview can be meaningful in multiple ways. First, it offers insight into patterns of argumentation, which can help uncover arguments that go unmentioned or are insufficiently conceptualized or defended in the literature. Second, it offers researchers, clinicians, ethicists, and sponsors who are dealing with specific questions regarding participant selection for FIH trials an overview of choices to consider, as well as highlighting considerations that could be relevant to critically reflect upon when making these choices. Since determining which participants should be selected for a specific FIH trial depends on numerous contingent variables and a normative evaluation of the identified reasons, the results of this review as such do not provide definitive a priori answers to guide participant selection. Nonetheless, this review can provide input for informed critical reflection. For instance, in a FIH trial of a novel drug, where both inclusion of healthy volunteers (HVs) and patients could offer valuable scientific insights, researchers might recognize the advantages of including either group and seek considerations to reflect upon in making a decision. Similarly, in cases where the target population consists of diverse populations, sponsors may struggle with whether they should ensure diversity from the outset or whether a more selective approach is warranted initially. In this case, critical reflection on the identified reasons for and against either option can facilitate informed decision-making. Finally, the results of this review can inform further normative inquiry, such as assessing what normative weight should be attributed to the reasons outlined in this review.

## Methods

A systematic review of reasons was conducted, following the methodology outlined by Stretch and Sofaer for conducting systematic reviews of argument-based literature [7]. Systematic reviews of reasons aim to provide a comprehensive overview of all reasons mentioned in the academic discourse on a bioethical topic by analysing and synthesizing the wide range of arguments and perspectives in the literature.

### Search strategy

Six online databases were consulted across various disciplines, including medicine (PubMed and Embase), philosophy (The Philosopher’s Index and PhilPapers), and multidisciplinary studies (Web of Science and Academic Search Premier). The decision to include these databases was made in consultation with an experienced librarian. A search strategy combining terms for (1) ‘first-in-human’, (2) ‘participants’, and (3) ‘reasons’ was used. For each term, multiple relevant publications were screened to identify relevant synonyms. The final search string, using Boolean operators to capture the three concepts and their relevant synonyms, was then translated to the various databases (Additional file 1). The search strategy was conducted on 05–06-2023 and updated on 14-05-2024 and 09-01-2025. Additionally, on 18-12-2024, relevant books and book chapters were identified through the library of Leiden University, using the search term ‘first-in-human’. The results were collected and deduplicated in EndNote.

### Inclusion criteria

Full-text publications discussing the ethics of participant selection for FIH trials that mentioned one or more reasons for or against including a participant (sub) group were included. Publications discussing the ethics of participant selection for clinical trials in general or clinical phase 1a/1b, 2, 3, 4, or 5 trials only were excluded, as were publications that did not mention any reason for or against including any particular participant group.

This review aims to provide a comprehensive overview of the reasons for and against including specific participant groups in FIH trials. Accordingly, we focus exclusively on the literature concerning FIH^2^ trials. Although all FIH trials are phase 1 trials, not all phase 1 trials qualify as FIH. While there is a significant body of literature on the ethics of phase 1 trials - some of which may be relevant to FIH trials - this review does not include that broader literature due to conceptual differences and practical constraints.

### Data extraction and analysis

The titles and abstracts of publications were screened in EndNote. Potentially relevant publications were then screened in full. In instances of doubt about inclusion, publications were discussed within the research team until a consensus was reached. The included publications functioned as the starting set for a snowballing process. Snowballing involved screening the references (backwards snowballing) and citations (forward snowballing) of included publications according to the same set of in- and exclusion criteria, to identify additional relevant literature. Subsequently, ATLAS.ti was used to code (1) the reasons for or against (2) a certain participant group in relation to (3) the research field the publication focussed on. All mentioned participant groups were categorized into six overarching categories: HVs, patients (general), patients with less advanced-stage diseases (LASD), patients with more advanced-stage diseases (MASD), vulnerable populations, and diverse participant groups.^3^ ‘Vulnerable’ populations were initially identified only when explicitly referred to as such in publications. However, following a review of the literature on vulnerability and team discussions, (sub)groups were also classified as a ‘vulnerable’ population if they could reasonably be perceived as such based on one of the three conceptions of vulnerability: (1) inherent, (2) categorical, or (3) contextual. All reasons for or against these groups were then included in the ‘vulnerable populations’ category, regardless of whether the (sub)groups were explicitly identified as vulnerable in the original publication. Reasons provided by authors functioned as ‘narrow reasons’, and if the same reason was mentioned in more than one publication, these were bundled together under the same narrow reason. The narrow reasons were then classified under six broader themes, the so-called ‘broad reasons’: non-maleficence, beneficence, scientific value, efficiency, respect for persons, and justice. In instances where a narrow reason could fit under multiple broad reasons, or it was unclear to which broad reason the narrow reason related most, discussions were held amongst the research team to determine the most appropriate broad reason.

### Quality appraisal

Currently, no methods exist to perform a formal quality appraisal for this methodology [7]. As such, the quality of included publications has not explicitly been assessed. Instead, we adopted the approach outlined by de Jongh et al., evaluating each included publication to determine the extent to which participant selection for FIH trials was discussed [8]. We differentiated between three categories: publications in which the participant selection for FIH trials is discussed briefly (e.g., a single paragraph devoted to the topic or one reason given somewhere), substantially (e.g., multiple paragraphs discuss the topic or many reasons are mentioned across the publication), or extensively (e.g., participant selection for FIH trials is the main topic of the publication). While a more substantial or extensive discussion does not necessarily indicate higher manuscript quality, it does provide some insight into how thoroughly authors have engaged with the topic and the degree of consideration they have given to the ethical challenges surrounding participant selection. For an overview of the characteristics of the included publications and the classifications, see Additional file 2.

## Results

**Fig. 1:**
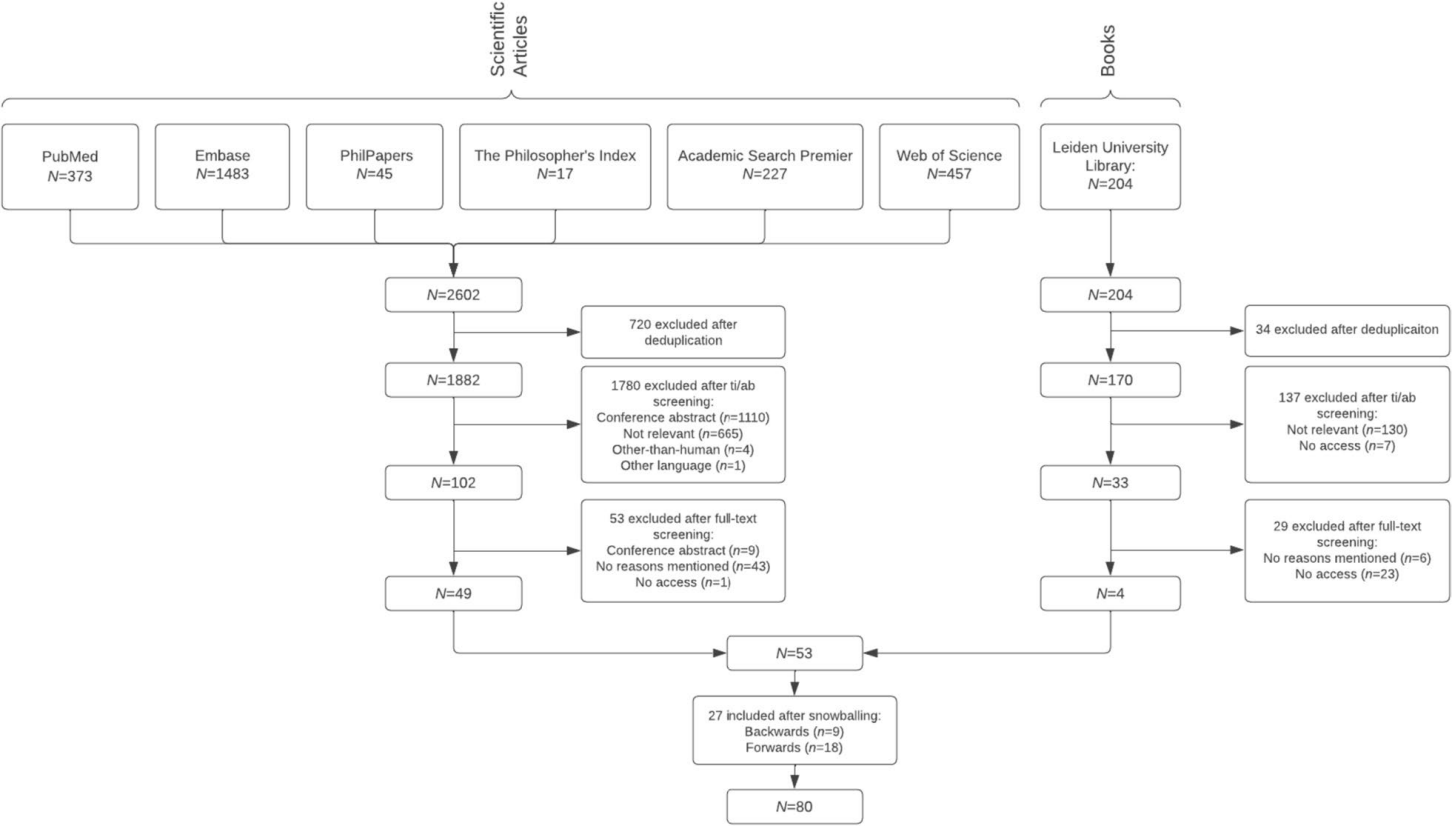
Flow chart of publication selection

**Fig. 2:**
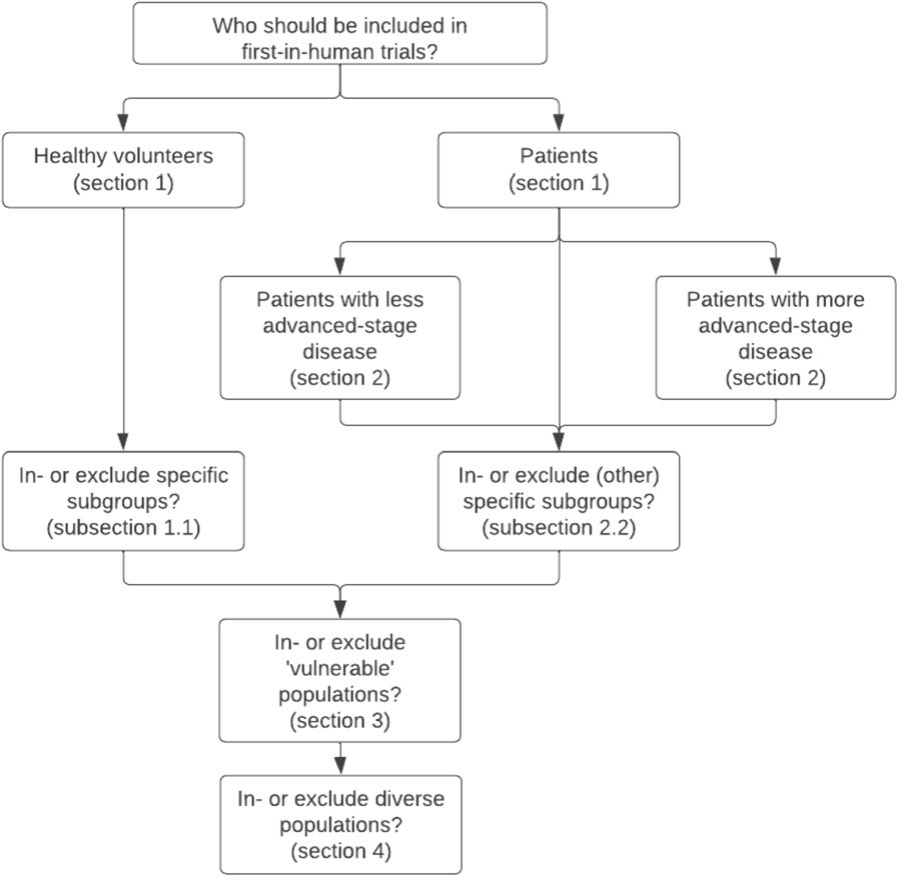
Flow chart of overall choices relevant to participant selection for FIH trials

The search strategy resulted in a total of 2602 publications and 204 books.^4^ After title/abstract screening, full-text screening, and snowballing, 80 publications were included for data extraction and analysis (Fig. 1). 181 reasons for and against the inclusion of the six potential participant groups were identified. The results are presented in this section, which is divided into four sections that reflect overall choices relevant to participant selection for FIH trials (Fig. 2). ”Should FIH trials be conducted with HVs or patients?” section addresses whether FIH trials should be conducted with HVs or patients, with "Reasons to in‑ or exclude specific subgroups of HVs" Subsection focussing specifically on the identified reasons for and against including specific subgroups of HVs. The "Should FIH trials be conducted with patients with LASD or MASD?" section explores whether FIH trials should be conducted with patients with LASD or MASD, with "Reasons to in‑ or exclude specific subgroups of patients" Subsection focussing on the reasons for and against including specific subgroups of patients. Sects. “Should FIH trials be conducted with vulnerable populations?” and “Should FIH trials be conducted with diverse participant groups?” explore reasons for and against conducting FIH trials with vulnerable populations and with diverse participant groups.

## Should FIH trials be conducted with HVs or patients?

The first relevant question when selecting participants for FIH trials is whether the trial should be conducted with HVs or patients. For an overview of the identified reasons to include HVs instead of patients, see Table 1, and for an overview of the identified reasons to include patients instead of HVs, see Table 2.

**Table 1:** Reasons to include HVs instead of patients (N = 33)

Narrow reason	N	Reference(s)
**Efficiency**		
* Including HVs*		
Could allow for faster and easier recruitment	6	[9, 11–14, 31]
Could lower trial costs	5	[9, 13, 14, 31, 32]
Could allow for exploring a wider range of doses within a shorter time frame	2	[9, 14]
Could improve the logistics of finding appropriate trial sites	2	[9, 14]
Could allow for studying the impact of age, gender, and ethnicity on PK alongside a SAD study	1	[9]
Could accelerate timelines of clinical development programs	1	[9]
Could allow for drug-free washout periods	1	[14]
Could allow for better protocol compliance	1	[14]
Could reduce the burden on drug suppliers	1	[14]
Could decrease drop-out rates	1	[14]
Could allow for closer safety monitoring	1	[14]
*Including patients*		
Could increase operational complications	2	[4, 14]
Could increase difficulties in, and costs of recruitment	1	[4]
Could allow for a wash-out period of non-trial medication	1	[13]
Could allow for the use of multiple trial sites	1	[14]
Could result in a longer trial duration	1	[14]
Could increase the incidence of non-compliance	1	[14]
**Scientific value**		
*Including HVs*		
Could lead to better-quality data	9	[4, 5, 9, 11, 13, 14, 16, 18, 28]
Could make it easier to assess the PK of a novel drug	3	[9, 11, 14]
Could increase the acquisition of useful data	2	[21, 28]
Could allow for a more impartial assessment of safety by including placebo subjects	1	[14]
Could help to inform a more appropriate starting dose in patients	1	[14]
* Including patients*		
Could affect the homogeneity of the data	1	[4]
Could complicate the interpretation of PK data	1	[14]
**Non-maleficence**		
*Including HVs*		
Could limit exposing patients to low, subtherapeutic doses	2	[9, 12]
Could prevent patients from forgoing a treatment known to be effective	1	[10]
Could decrease the number of participants needed	1	[11]
* Including patients*		
Could worsen disease development	2	[4, 5]
Could lead to poor outcomes	1	[13]
**Beneficence**		
*Including HVs*		
Could decrease the risk of (serious) adverse effects	2	[5, 11]
Could provide access to health services they would otherwise not qualify for or be able to afford	1	[4]
**Respect for persons**		
* Including patients*		
Could lead to a greater lack of comprehension or disregard for the information conveyed	2	[4, 29]
Could lead to including participants who are vulnerable to undue influence or coercion	1	[33]

**Table 2:** Reasons to include patients instead of HVs (N = 20)

Narrow reason	N	Reference(s)
**Non-maleficence**		
*Including HVs*		
Could expose them to disproportional risks	15	[4, 5, 9–11, 14–23]
Could lead to participants not receiving compensating health benefits	12	[4–6, 9, 11, 12, 15, 21, 24–27]
Could cause (serious) adverse effects that are deemed unacceptable or intolerable	3	[9, 13, 17]
Could lead to less clarity on the long-term effects of the intervention	1	[4]
* Including patients*		
Could expose them to lower relative risk	4	[10, 13, 17, 19]
Could decrease the number of participants necessary	1	[17]
**Beneficence**		
* Including patients*		
Could allow for direct benefits	4	[13, 14, 24, 27]
Could avoid delays in offering patients the opportunity to benefit from a potential cure	1	[14]
**Respect for persons**		
* Including HVs*		
Could lead to including participants who withhold information about their health status if monetary incentives are involved	4	[4, 5, 21, 35]
Could lead to including participants who could not be able to give genuine informed consent	4	[21, 24, 26, 34]
Could lead to including participants who are vulnerable to exploitation	3	[5, 21, 24]
Could lead to including participants who are not able to understand the risks they are taking	1	[24]
* Including patients*		
Could increase the autonomy of family caregivers	1	[18]
Could increase their moral right to make autonomous decisions	1	[36]
**Scientific value**		
*Including HVs*		
Could lead to insights that cannot be translated to or do not apply to patients	12	[9, 11, 13, 14, 17, 21, 22, 25, 27, 29–31]
*Including patients*		
Could generate more better-quality data	4	[5, 14, 16, 17]
Could allow for early evidence of target violation and clinical response	1	[14]
**Efficiency**		
*Including HVs*		
Could delay overall timelines	1	[32]
Could increase operational complexity	1	[32]
*Including patients*		
Could reduce costs, as PD is measurable and able to show early indicators of activity	1	[14]

### Non-maleficence^5^

Non-maleficence towards patients was considered a reason to include HVs in FIH trials instead of patients. Authors note that recruiting HVs as participants for FIH trials could alleviate some of the burden on patients [9–12]. For instance, patients would be spared from exposure to low, subtherapeutic doses [9, 12], would not have to forgo an effective treatment due to participating in FIH research [10], and would not have to carry the overall emotional and physical burdens of being involved in FIH research [11]. Due to their stable health status, HVs could also experience fewer (serious) adverse effects than patients [5, 11]. On the other hand, including patients in FIH trials could exacerbate their existing medical conditions due to drug toxicities [4], lead to poor outcomes given their fragile state of health [13], or worsen their disease altogether [5].

Most reasons authors gave to include patients rather than HVs relate to the non-maleficence of *HVs*. Many authors expressed that HVs could face a higher relative risk [4, 5, 9–11, 14–23] while patients could face a lower relative risk [10, 13, 17, 19]. While some authors have argued that HVs can derive direct or indirect benefits from participating in FIH research, many others contend that, unlike patients, they are unable to receive potentially compensating health benefits [4–6, 9, 11, 12, 15, 21, 24–27]. Additionally, authors have raised concerns about the long-term effects of interventions, which may not always be (directly) measurable in HVs [4]. There are also worries about the possibility of the intervention causing (serious) adverse effects that would be deemed unacceptable or intolerable for HVs [9, 13, 17]. Finally, concerns about the non-maleficence of HVs were brought up for FIH trials of Active Medical Implanted Devices (AMID). Since safe stimulation levels in HVs do not necessarily represent the effective stimulation needed to treat symptoms in patients, recruiting only patients who might require the potential treatment would minimize participant numbers; reducing potential harm [17].

### Beneficence

Other reasons for including HVs in FIH trials were given based on beneficence. These encompass potential indirect benefits that HVs could gain from participating in FIH research, such as accessing health services they would otherwise not be qualified for or able to afford [4].

However, authors presented more reasons for including patients based on beneficence. Compared to HVs, patients could receive compensating health benefits [13, 14, 24, 27] and derive *indirect* benefits from participation in FIH research [4]. For instance, in the form of improved healthcare services [4]. Authors furthermore argued that by including patients in FIH trials, delays in offering patients a potential cure could be avoided [14].

### Scientific value

Authors emphasize that including HVs instead of patients in FIH trials could enhance the quality of acquired data [4, 5, 9, 11, 13, 14, 16, 18, 21, 28], as data is less likely to be compromised by co-morbidities [4, 5, 9, 11, 14] or co-administered drugs [4, 5, 9, 13, 16], and may be more homogeneous [21]. In contrast, authors worry that by including patients, data could be confounded by co-morbidities and concomitant medications [14] and note that the homogeneity of the data could be prejudiced [4] since there is more diversity in patients’ medical statuses than in those of HVs. Furthermore, authors note that data obtained from HVs could aid in determining an appropriate starting dose [14] and that a more impartial evaluation of safety could be achieved by including placebo subjects [14]. Not only a drug’s safety but also its pharmacokinetics (PK) could be better assessed [9, 11, 14] since it is more acceptable to intensively collect PK blood samples in HVs than it is in patients [9]. Lastly, including HVs in FIH trials enables more extensive confinement, facilitating the collection of comprehensive PK data throughout the day, thereby allowing researchers to collect detailed information on how the body processes the drug [14].

Despite these reasons, authors also presented reasons based on scientific value to include patients instead of HVs. For instance, authors expressed concerns about the usefulness of data obtained from HVs [9, 11, 13, 14, 17, 21, 22, 25, 27, 29–31]. Most worries relate to potential differences in pharmacodynamics (PD) measures [9, 11, 13, 14, 25, 29, 31] and PK properties [9, 14] between HVs and patients, as well as differing target-related safety measures [14, 17]. Furthermore, in some cases, the target of the investigational intervention might only exist in patients [21, 30]. Some interventions might also require long-term use to measure potential acclimatization or tolerance aspects, making the inclusion of HVs more complicated [27]. Data acquired from patients on the other hand could be more valuable and informative [5, 14, 16, 17]. This heightened value is linked to the critical role of PK in patients [5, 14], which could enable the assessment of early indicators related to target violation and clinical response [14]. Moreover, data obtained from patients could provide knowledge that is more directly applicable to improving therapies [17].

### Efficiency

Most reasons to include HVs instead of patients for FIH trials are related to trial efficiency. Authors note that including HVs could make recruitment faster and easier [9, 11–14, 31]. This is in part because recruitment could be more predictable for HVs [13], and because non-eligible subjects in this group are rare [11]. While including patients could complicate and increase the cost of the recruitment process [4], trials could be less expensive, with lower costs per subject, when conducted with HVs [9, 13, 14, 31, 32]. Furthermore, including HVs could require fewer trial sites [14], while the broader range of eligible trial site options for this group allows for more flexibility in site selection [21]. Additionally, authors mention that including HVs could accelerate the timelines of clinical development programs [9, 14]. This is because a wider range of doses can be explored within a shorter timeframe [9, 14], and researchers can concurrently study the impact of age, gender, and ethnicity on PK alongside dose-finding [9]. Including patients could increase the chance of operational complications during the trial [4, 14] and allow for a wash-out period of non-trial medication [13]. In contrast, delays in the trial process could be kept to a minimum by including HVs as drug-free wash-out periods are unnecessary [14]. Another advantage of including HVs is that monitoring the safety of the intervention or drug could be more feasible [14]. Furthermore, several reasons highlight the *attributes* of HVs compared to patients, suggesting that HVs could exhibit lower drop-out rates [14] and have better protocol compliance [14]. Lastly, one publication noted that by including HVs the burden on drug suppliers could be somewhat alleviated [14].

However, there are also reasons based on efficiency to include patients instead of HVs. One publication noted that including patients allows for the measurement of PD values, offering early signs of activity, which could reduce trial costs [14]. HVs cannot provide signs of activity through PD measures because they do not have the target of the disease. The publication furthermore asserted that including patients in FIH research could offer a competitive advantage in the regulatory environment of the United States by streamlining development [14]. Finally, one publication noted that in practice, including HVs in FIH trials might delay overall timelines instead of accelerating them [32]. Furthermore, operationalization may be complicated [32] because including HVs in FIH trials, instead of testing immediately in patients, might require an extra trial, which would in many cases be executed by a different department.

### Respect for persons

Finally, authors highlighted reasons concerning respect for persons and warned that patients could experience a higher degree of difficulty in comprehending information that is provided to them, as well as be more inclined to discount information [4, 29], particularly when they find themselves in a desperate state of mind [29]. Additionally, patients may be vulnerable to undue influence or coercion, especially in cases where patients’ family members have different attitudes towards or understandings of the intervention [33].

However, there are also reasons to include patients instead of HVs based on respect for persons. Firstly, authors highlight that HVs might not fully understand the risks they are taking by participating in FIH research [24] and cannot give *genuine* informed consent [21, 24, 26, 34]. Worries around consent are exemplified when monetary incentives are involved [21, 34]. Authors also note that HVs could be vulnerable to exploitation [5, 21, 24], for instance, due to monetary incentives [5, 21], hierarchical relationships [21], and/or because they could not be able to give genuine informed consent [24]. Monetary incentives could also encourage HVs to conceal health information that might get them removed from the trial [4, 5, 21, 35]. There are also specific reasons to *include patients* based on respect for persons. One publication particularly underscored the significance of autonomy for patients with decision-making capacity, arguing that patients should be able to decide for themselves if they want to participate in FIH research [36]. A final consideration pertains to the autonomy of patients’ family caregivers. In their work, Hug and Herméren have argued that by involving patients in FIH trials, their family caregivers could regain a greater sense of autonomy in their lives [18].

### Reasons to in- or exclude specific subgroups of HVs

If a FIH trial is conducted with HVs, the question arises whether there are reasons to include and exclude specific *subgroups* of HVs. For an overview of the identified reasons for including specific subgroups of HVs see Table 3. For an overview of the identified reasons against including specific subgroups of HVs see Table 4.

**Table 3:** Reasons to include specific subgroups of HVs (N = 2)

Narrow reason	N	Reference(s)	Subgroup
**Beneficence**			
Could delay or prevent the onset of disease	1	[15]	Individuals at risk
Could increase benefits	1	[37]	Special forces personnel

**Table 4:** Reasons to exclude specific subgroups of HVs (N = 4)

Narrow reason	N	Reference(s)	Subgroup
**Non-maleficence**			
Could be exposed to additional safety risks	4	[4, 5, 24, 28]	HVs who participate in multiple trials, have specific habits or undisclosed physical conditions
Could lead to unnecessary treatment	1	[15]	Individuals at risk
Could expose future patients to unforeseen safety and efficacy issues	1	[24]	HVs who participate in multiple trials, have specific habits or undisclosed physical conditions
**Scientific value**			
Could compromise the quality of the data	3	[5, 21, 24]	HVs who participate in multiple trials, have specific habits or undisclosed physical conditions

### Beneficence

Two reasons were offered for including specific subgroups of HVs based on beneficence. First, authors note that in some cases, including individuals at risk of developing the targeted disease could provide *direct* benefits, namely by delaying or preventing the onset of disease, thereby improving overall health outcomes [15]. In the context of FIH trials of gene therapy for military enhancement, authors noted that including special forces personnel, who are involved in high-risk missions, could confer (larger) direct benefits through optimizing human performance [37].

### Non-maleficence

Authors provided several reasons against including specific subgroups of HVs based on non-maleficence. For instance, some authors argued that including HVs at risk of the targeted disease could lead to ‘‘unnecessary treatment’’ (p.11) since not all at-risk individuals will develop the disease [15]. Reasons against including HVs participating in multiple trials (simultaneously or with insufficient drug-free wash-out periods) were also presented. Authors noted that these HVs could be more vulnerable to harm [4, 5, 24], for instance, due to potential drug interactions [24]. Due to the compromising effects of drug interactions on data validity, their participation could also expose future patients to unforeseen safety and efficacy issues [24]. HVs with specific habits (e.g., smoking or alcohol and drug consumption) or undisclosed physical conditions (e.g. psychological or neurological conditions) could also be more prone to harm [4, 5, 28] since they could be more inclined to conceal health information out of fear of being excluded from the trial [28].

#### Scientific value

Lastly, one concern with regard to scientific value was raised specifically regarding HVs who participate in multiple trials at the same time or rapidly precede one another. Data obtained from these HVs could be compromised due to their exposure to numerous different interventions and/or medications [5, 21, 24].

## Should FIH trials be conducted with patients with LASD or MASD?

If a FIH trial should be conducted with patients, a subsequent question is whether the trial should be conducted with patients with less advanced-stage disease or more advanced-stage disease. In this section, the reasons for and against including patients with LASD versus MASD are presented. For an overview of the reasons to include patients with LASD instead of MASD see Table 5. For an overview of the reasons to include patients with MASD instead of LASD see Table 6.

**Table 5:** Reasons to include patients with LASD instead of MASD (N = 36)

Narrow reason	N	Reference(s)
**Beneficence**		
* Including patients with LASD*		
Could allow participants to receive direct or indirect benefits	13	[5, 6, 15, 19, 39, 42, 45, 47, 49, 56, 58, 62, 63]
Could allow for fewer procedure- and disease-related complications	2	[19, 51]
Could prevent (long-term) severe adverse effects	2	[15, 19]
Could halt the progression of their disease	2	[15, 47]
Could allow them access to a possibly superior treatment option	1	[26]
Could lead to including participants who have better safety records in other trials	1	[26]
Could allow participants to turn to proven therapies at a later time point	1	[44]
Could allow for a better risk–benefit balance based on pre-clinical data	1	[50]
**Non-maleficence**		
*Including patients with MASD*		
Could lead to including participants who have less to gain	11	[4, 6, 10, 15, 19, 20, 38–42]
Could cause participants to suffer serious losses	4	[4, 5, 16, 17]
Could worsen disease development	4	[10, 15, 17, 36]
Could allow for more procedure- and disease-related complications	2	[19, 43]
Could lead to including participants for whom time commitments and schedule disruption might be more burdensome	2	[46, 47]
Could harm participants due to the lack of access to their usual treatment which alleviates some of their symptoms	1	[18]
Could cost participants their eligibility status for a subsequent trial that may hold therapeutic benefits	1	[21]
Could not be ideal for medical reasons	1	[29]
Could lead to including participants who are less likely to respond to the intervention	1	[45]
Could increase risks of (severe) adverse effects	1	[44]
**Respect for persons**		
* Including patients with MASD*		
Could lead to including participants who are more susceptible to therapeutic misconception	15	[4, 5, 16, 26, 29, 33, 39, 46, 47, 50, 64–68]
Could lead to including participants who are affected in their capacity to give informed consent	12	[5, 17, 21, 26, 36, 39, 42, 46, 47, 54, 61, 69]
Could interfere with a healthy dying process	1	[26]
Could lead to including participants who are vulnerable to exploitation	1	[29]
*Including patients with LASD*		
Could lead to including participants who can give sufficiently informed consent	4	[16, 21, 39, 46]
Could lead to including participants who are less susceptible to therapeutic misconception	1	[5]
Could lead to including participants whose moral objectives align more with those of the investigators	1	[26]
**Scientific value**		
*Including patients with MASD*		
Could decrease the data quality	14	[17, 18, 22, 26, 29, 33, 36, 39, 43, 44, 46, 56–58]
Could make it impossible to monitor long-term effects	2	[10, 18]
Could jeopardize the (initial) success of the field	2	[41, 47]
Could be less likely to show the desired biological responses	2	[42, 46]
* Including patients with LASD*		
Could allow for better-quality data	4	[5, 44, 58, 64]
Could enable the evaluation of long-term effects	1	[5]
Could allow for easier assessment of side effects and safety	1	[44]
**Efficiency**		
*Including patients with MASD*		
Could lead to including participants for whom it may be challenging to visit during office hours	2	[13, 49]
Could lead to including participants who have additional comorbidities that may require management with prescription and non-prescription medication	1	[14]
Could greatly reduce the pool of potential participants	1	[36]
*Including patients with LASD*		
Could reduce the number of participants needed to reach statistical significance	1	[49]

**Table 6:** Reasons to include patients with MASD instead of LASD (N = 30)

Narrow reason	N	Reference(s)
**Non-maleficence**		
*Including patients with LASD*		
Could lead to including participants who face a higher relative risk	11	[5, 15, 18, 21, 23, 26, 39, 40, 45, 46, 48]
Could lead to including participants who still have alternative treatment options	4	[15, 21, 39, 44]
Could cause them to miss out on a more effective intervention	3	[11, 17, 18]
Could worsen disease development	2	[15, 40]
Could affect their quantity or quality of life in case of (serious) adverse effects	2	[18, 19]
Could affect the chance of spontaneous recovery	2	[49, 50]
Could allow participants to benefit less	1	[10]
Could lead to unnecessary treatment	1	[40]
Could have a higher chance of developing (serious) adverse effective over the long term	1	[46]
* Including participants with MASD*		
Could lead to including participants who have the least to lose	21	[5, 15–19, 29, 33, 38, 40, 45, 47, 49, 51–58]
Could lead to including participants who face a lower relative risk	14	[10, 18, 19, 21, 26, 29, 36, 39, 45, 46, 56, 59–61]
Could allow for lower long-term risks	4	[10, 29, 44, 46]
Could lead to including the only participants for whom the public health risks associated with the possibility of zoonotic infections can be justified	2	[36, 42]
Could prevent other patients from forgoing a treatment known to be effective	1	[10]
Could protect other patients against unjustified and uncertain harms of unproven interventions	1	[44]
Could not lose a chance of survival by participating in FIH research	1	[58]
Could lead to including the only participants to whom the safety risks surrounding procreation can be justified	1	[59]
**Beneficence**		
*Including patients with MASD*		
Could allow participants to receive direct or indirect benefits	7	[4, 5, 10, 15, 23, 46, 61]
Could treat debilitating symptoms	1	[43]
Could increase social value	1	[49]
Could lead to including participants who have the most to gain	1	[53]
Could lead to more effectiveness of the treatment	1	[56]
Could decrease the burden of life-long monitoring on patients with LASD	1	[59]
**Respect for persons**		
* Including patients with MASD*		
Could lead to including participants who are less susceptible to therapeutic misconception	3	[19, 21, 49]
Could allow for including participants who can give acceptable informed consent	1	[21]
**Scientific value**		
*Including patients with MASD*		
Could allow for better-quality data	1	[49]
Could avoid dramatic outcomes; decreasing trust in the field	1	[38]
**Justice**		
* Including patients with MASD*		
Could present them with the last chance to participate in research that is potentially beneficial to them	7	[15, 19, 36, 62, 64, 70, 71]
Could stimulate equal opportunity for access	1	[67]
**Efficiency**		
* Including patients with LASD*		
Could lead to including participants who must combine trial participation with a daytime job	1	[13]

### Non-maleficence

When it comes to non-maleficence, authors provided several reasons to include patients with LASD instead of MASD. Many authors suggested that patients with MASD have the least to gain from the intervention [4, 6, 10, 15, 19, 20, 38–42] and could be more susceptible to procedure and disease-related complications [19, 43], particularly those with numerous co-morbidities [43]. Furthermore, this group could face an increased risk of (severe) side effects and adverse reactions (44). Within the context of stem cell research, authors asserted that patients with MASD might ‘‘…not be the ideal choice for medical reasons’’ (p.1083) [29]. Other concerns were raised about the well-being of patients with MASD, noting the potential lack of response to the intervention [45] and worsening of the disease [10, 15, 17, 36]. Authors also highlighted that participating in FIH research could harm patients with MASD, either by temporarily depriving them of their usual treatment, which might alleviate some of their symptoms [18] or by causing them to lose their eligibility status for a subsequent trial that may hold therapeutic benefits [19]. Some publications specifically highlighted the importance of considering the fragile state of patients with MASD. For instance, authors noted that time commitments and schedule disruption could be more burdensome to these patients [46, 47]. Furthermore, some authors emphasized that despite their advanced condition, these patients could still experience significant losses, placing a duty on researchers to protect them from potentially compromising their limited or poor quality of life by participating in FIH research [4, 5, 16, 17, 46].

However, despite these reasons, there are also many reasons to include patients with MASD based on the non-maleficence of patients with LASD. Authors expressed that by participating, patients with LASD could be exposed to disproportional risks [5, 15, 18, 21, 23, 26, 39, 40, 45, 46, 48] while alternative treatment options are still available to them [15, 21, 39, 44]. This implies that their participation in FIH research could potentially deny them access to a more effective intervention [11, 17, 18]. However, other authors point out that these patients could generally still explore proven treatment options *after* their research participation. Additional concerns revolve around the potential *consequences* of including patients with LASD in FIH research. Authors note that including this group could lead to unnecessary treatment [40], for instance in cases where there is still a possibility of spontaneous recovery [49, 50]. Including these patients could also worsen the disease development [15, 40]. In case of complications or (serious) adverse effects, including this group could negatively affect patients’ quantity or quality of life [18, 19] by shortening their life expectancy [18] or reducing the number of years during which they experience a high quality of life [19]. Relatedly, one publication noted that these patients could have a higher chance of developing long-term (serious) adverse effects as they are more likely to survive for a longer time than patients with MASD [46]. On the other hand, many authors indicated that including patients with MASD in FIH research might be justifiable because these patients could have the least to lose [5, 15–19, 29, 33, 38, 40, 45–47, 49, 51–58], and their risk–benefit balance could be proportional [10, 18, 19, 21, 26, 29, 36, 39, 45, 46, 56, 59–61]. This is not only because the trial’s risk–benefit balance is (more) justifiable [21, 36, 39, 61] but also because they have no alternative treatment options (anymore) [18, 21, 29, 39, 56]. Relatedly, authors argued that these patients would not lose a chance of survival by participating in FIH research [58]. Within the context of xenotransplantation, authors noted that the public health risks associated with the possibility of zoonotic infections could only be justified for patients with MASD [36, 42].^6^ Other authors in the field of xenotransplantation assert that the extra safety risks surrounding procreation provide an argument for including patients with MASD, as these risks could only be justified for patients to whom the xenograft would be life-saving [59]. Other publications note that patients with MASD could be suitable for FIH research where there are concerns about the intervention’s long-term consequences since these patients are expected to live for fewer years [10, 29, 44, 46]. Moreover, performing FIH research in this group could diminish the burden of long-term, or within the field of xenotransplantation, lifelong monitoring [59]. Finally, by including patients with MASD, other patient groups could be protected against unjustified and uncertain harms of exposure to unproven interventions [44].

### Beneficence

Most reasons for including patients with LASD are based on beneficence. Authors highlighted that these patients could experience both direct and indirect benefits from participating [5, 6, 15, 19, 39, 42, 45–47, 49, 56, 62, 63]. The intervention could furthermore enhance health outcomes by impeding disease progression [15, 47] and offer patients a potentially superior treatment approach [26]. Authors also suggest that patients with LASD could be less susceptible to procedure- and disease-related complications [15, 19, 51], including (serious) adverse effects [15, 19]. Moreover, these patients could still turn to proven standard therapies if needed [44]. Additionally, within the context of stem cell research, including patients with LASD could enhance the social value of FIH research since increased health outcomes in this group could benefit society [49]. Finally, two reasons were based on previous evidence. Authors noted that patients with LASD possess a better safety record based on prior FIH trials [26] and could have a more favourable risk–benefit balance based on pre-clinical data [50].

Other reasons were given for patients with MASD. Authors noted that these patients could receive direct or indirect benefits from participating in FIH research [4, 5, 10, 15, 23, 61], such as psychological benefits, greater access to healthcare resources, prolonged life or improved quality of life [23]. Furthermore, one publication asserted that patients with MASD could ‘‘have the most to gain from any trial outcomes’’ (p.6) [53]. Within the context of deep brain stimulation, including patients with MASD could lead to the treatment of some of their debilitating symptoms [43], while within the context of cardiovascular medicine evidence suggests that the intervention is more effective in patients with MASD [56].

### Scientific value

Authors also presented reasons for including patients with LASD based on scientific value. To start, the quality of data obtained from patients with MASD could be lower since there are more confounders in this patient group [17, 18, 22, 26, 29, 33, 36, 39, 43, 44, 46, 56–58]. As a result, these patients could be less likely to display, and it could be harder to measure, the desired biological response [42, 46]. For instance, in the context of cardiac xenotransplantation, patients with MASD have a high risk of dying irrespective of whether they participate in FIH research or not [42]. Additionally, it could be more challenging to measure the long-term effects of the intervention [10, 18], given that these patients are expected to live shorter than patients with LASD. Within the context of xenotransplantation, enrolling patients with MASD, who generally have more co-morbidities, could also jeopardize the initial success of the field [41] as these patients could attain less significant benefits from the intervention due to their fragile state of health. At the same time, the data obtained from patients with LASD could be of better quality as there are fewer confounders in this group [5, 44, 58, 64]. This reduced complexity streamlines the assessment of potential side effects and intervention safety [44]. Finally, the longer survival expectancy within this group could allow for a more comprehensive evaluation of long-term effects [5].

However, authors also noted that patients with MASD could generate better-quality data [49] as therapeutic benefits could be clearer and more readily quantifiable, particularly for patients with chronic diseases, who could provide a stable environment to assess the safety of the intervention. By enrolling patients with MASD, who might have a more proportional risk–benefit balance, investigators could also avoid dramatic health outcomes, such as serious adverse effects or deaths, which could result in crises of confidence and decrease trust in the respective fields [38, 47].

### Efficiency

Including patients with LASD in FIH trials might also benefit trial efficiency. For instance, patients with MASD could have additional co-morbidities that may require management with prescription and/or non-prescription medication [14]. Furthermore, patients with MASD may require more care and have more (family) caregivers. If these caregivers have daytime jobs, this group could experience difficulties with visits during office hours [13]. Opting to include patients with MASD could also greatly reduce the pool of potential participants [36], whereas by including patients with LASD, the number of participants needed to reach statistical significance could be reduced [49].

One reason was presented to include patients with MASD based on trial efficiency: patients with LASD could have more difficulties with trial participation because they could need to balance participating in FIH research with daytime employment [13].

### Respect for persons

Regarding respect for persons, authors indicated that patients with MASD could be more susceptible to therapeutic misconception [4, 5, 16, 26, 29, 33, 39, 46, 47, 50, 64–68] and be affected in their capacity to provide informed consent [5, 17, 21, 26, 36, 39, 42, 46, 47, 54, 61, 69], whereas patients with LASD could be able to provide informed consent [16, 21, 39, 46] and be less susceptible to therapeutic misconception [5]. The moral objectives of patients with LASD could furthermore align more with those of the investigators than the moral objectives of other patient groups [26]. Lastly, one publication asserted that recruiting patients with MASD in FIH research could interfere with a healthy dying process [26]. Another aspect considered was this group’s vulnerability to exploitation [29], which was considered a reason to refrain from including them in FIH research to protect them against potential exploitation.

Contrary to the view of some authors, three publications mentioned that enrolling patients with MASD could *reduce* rather than increase the risk of therapeutic misconception [19, 21, 49]. Patients with MASD have lived with their diagnoses for a longer time and are more experienced with their disease. This could allow them to make better-informed decisions about trial participation than recently diagnosed patients. This is particularly applicable to patients with chronic diseases [21, 49] or older patients [19]. Furthermore, authors noted the importance of autonomy in this patient group, arguing that patients with MASD can give sufficiently informed consent [21] as their consent is accepted and valid in other contexts as well.

### Justice

Lastly, authors presented reasons to include patients with MASD based on justice. Since patients with MASD may have no alternative treatment options (anymore), and their inclusion in a FIH trial could represent a final opportunity for these patients to engage in potentially beneficial research, it could be just to include them in FIH research [15, 19, 36, 62, 64, 70, 71]. Including patients with MASD might furthermore be considered because it could stimulate equal opportunity of access to FIH trials across patient groups [67].

## Reasons to in- or exclude specific subgroups of patients

Regardless of whether patients with LASD or MASD should be included, authors have also provided several reasons for including and excluding specific *subgroups* of patients. For an overview see Tables 7, 8.^7^

**Table 7:** Reasons to include specific patient subgroups (N = 5)

Narrow reason	N	Reference(s)	Subgroup
**Beneficence**			
Could allow for indirect benefits	2	[4, 13]	Patients who have no job or live on their own
Could allow for little or no cognitive dysfunction as a result of the intervention	1	[43]	Younger patients
Could allow for a faster recovery	1	[43]	Younger patients
Could enhance post-transplant resilience	1	[72]	Patients with a high level of social support
**Respect for persons**			
Could provide them a potential treatment option	2	[36, 72]	Patients who have moral or religious objections to other treatment options

**Table 8:** Reasons to exclude specific patient subgroups (N = 3)

Narrow reason	N	Reference(s)	Subgroup
**Non-maleficence**			
Could lead to including participants who need more revision rates	1	[40]	Younger patients
Could lead to participants who face a higher relative risk	1	[10]	Older patients
**Scientific value**			
Could negatively affect the quality of the data	1	[46]	Older patients

### Beneficence

Firstly, within the context of deep brain stimulation, authors provided reasons to include younger patients based on beneficence. Some authors noted that these patients could experience little to no cognitive dysfunction from the intervention [43] and recover faster [43] than other patients. Patients with a good support system are another subgroup that could have distinct advantages compared to other patient (sub)groups. Within the context of xenotransplantation, these patients may be included in FIH trials, instead of other patient groups, because their social support system could bolster their recovery after the transplant [72]. Lastly, authors argued that including patients who are unemployed and/or live alone can in some cases provide (additional) indirect benefits to them through added social interaction [11].

### Respect for persons

Within the context of xenotransplantation, authors argued that patients who have moral or religious objections to receiving an allograft (human donor heart) could qualify for a xenograft (pig donor heart) because patients should have the right to choose which treatment they do and do not want [36, 72].

### Non-maleficence

Two reasons were provided against including younger patients on the basis of non-maleficence. Authors noted that within the context of orthopaedics, younger patients at risk of requiring a knee prosthesis would face higher revision rates for such prostheses. Therefore, including younger patients in FIH research could result in a need for more revision procedures throughout their lives [39]. On the other hand, including older patients in FIH research, compared to young and middle-aged patients, could lead to the inclusion of a subgroup that faces a higher relative risk. For instance, in the context of stem cell research for Parkinson’s Disease, patients older than 70 face a higher neurosurgical risk [10].

### Scientific value

One reason was provided against including older patients. Authors noted that older patients often have more co-morbidities which can complicate the analysis of safety data [46].

## Should FIH trials be conducted with vulnerable populations?

Within the literature, special attention is paid to the question of whether specific ‘vulnerable’ populations—such as people with psychiatric conditions, newly diagnosed or disabled patients, (pregnant) women, and children—should be included in FIH research. For an overview of the reasons to exclude vulnerable populations see Table 9. For an overview of the reasons to include vulnerable populations see Table 10.

**Table 9:** Reasons to exclude vulnerable populations (N = 23)

Narrow reason	N	Reference(s)	Subgroup
**Non-maleficence**			
* Including vulnerable populations*			
Could lead to including participants who face a higher relative risk	3	[15, 34, 36]	Pregnant women [34] and children [15, 36]
Could lead to including participants who face additional risks	2	[36, 43]	Patients who suffer from depression [43], are at risk of dying by suicide or have a psychiatric disorder [36]
Could lead to including participants who experience more (serious) adverse effects	1	[11]	Women
Could lead to unnecessary treatment	1	[15]	Children
Could lead to including participants who still have alternative treatment options	1	[15]	Children
Could lead to including participants for whom it would be more burdensome to undergo long-term follow-ups	1	[15]	Children
Could lead to participants being negatively affected in their well-being	1	[43]	Patients who suffer from extreme anxiety
Could lead to including participants who would not significantly benefit from the intervention	1	[41]	Patients with an unstable psychiatric disease, inadequate caregivers or social support, patients with a history of poor compliance
Could lead to participants suffering greater long-term costs	1	[55]	Less advantaged patients
Could lead to participants suffering greater lifestyle difficulties	1	[55]	Less advantaged patients
**Scientific value**			
Could lead to difficulties in distinguishing between spontaneous recovery or recovery attributed to the intervention	2	[20, 22]	Newly diagnosed or disabled patients
Could lead to challenges in providing the necessary conditions to ensure high technical standards and the safety of research participants in low-income countries	2	[30, 46]	Patients from low-income countries [30], patients from low- and middle-income countries [46]
Could lead to participants who have more undiagnosed diseases and untreated morbidities affecting the quality of the data	1	[30]	Patients from low-income countries
Could jeopardize the generalizable knowledge obtained from the trial	1	[36]	Patients at risk of dying by suicide
Could confound efforts to fairly evaluate the procedure’s safety and efficacy	1	[41]	Patients with an unstable psychiatric disease, inadequate caregivers or social support, or patients with a history of poor compliance
**Efficiency**			
Could result in (more) cultural obstacles	1	[30]	Patients from low-income countries
Could negatively affect pre- and post-operative measures	1	[43]	Patients who suffer from depression
Could result in setbacks in the long term	1	[49]	
**Respect for persons**			
Could be affected in their capacity to give informed consent	17	[10, 15, 17, 20, 33–35, 39, 41, 46, 55, 59, 65, 74, 76, 79, 80]	Patients who are cognitively impaired [10, 33, 35, 39, 55, 79, 80], newly diagnosed or disabled patients [10, 20, 41, 65, 76] children [15, 17, 34, 35, 55, 59, 74], patients with a mental disability [55] and parents of dying infants [46]
Could be vulnerable to exploitation	5	[4, 28, 30, 35, 49]	Newly diagnosed or disabled patients [49], HVs with low incomes [4, 28, 35], patients from low-income countries [30]
Could be more susceptible to therapeutic misconception	5	[10, 20, 49, 63, 64]	Newly diagnosed or disabled patients
Could be vulnerable to undue influence	2	[49, 76]	Newly diagnosed or disabled patients
**Justice**			
Could be unfair due to their status as being vulnerable	1	[10]

**Table 10:** Reasons to include vulnerable populations (N = 20)

Narrow reason	N	Reference(s)	Subgroup
**Justice**			
*Including vulnerable populations*			
Could present them with the last chance to participate in research that is potentially beneficial to them	3	[6, 70, 75]	Children
Could reduce a culture of dependency on developed countries	1	[30]	Patients from low-income countries
Could increase economic activity by encouraging research into more innovative products	1	[30]	Patients from low-income countries
Could facilitate healthcare infrastructure development	1	[30]	Patients from low-income countries
Could drive capacity building for local ethical review	1	[30]	Patients from low-income countries
Could increase equitable distribution of risk and benefit in populations of prospective beneficiaries	1	[73]	
Could give them a voice	1	[74]	Parents and children
**Beneficence**			
Could allow them to benefit more from future interventions	8	[17, 34–36, 46, 73–75]	Children [17, 36, 74, 75], patients from low- and middle-income countries [46]
Could lead to including participants who would specifically benefit from the intervention	8	[36, 42, 58, 70, 75–78]	Patients with acute SCI [76], infants with complex congenital heart disease [36, 42], younger children (under 12) [58], children [70, 75, 77] and prepubertal boys [78]
Could lead to participants who would be able to enjoy the benefits of the intervention for the longest period of time	2	[15, 74]	Children
Could prevent serious (future) complications	1	[15]	Children
Could lead to including participants who would suffer less damage to the body	1	[15]	Children
Could increase psychological benefits	1	[58]	Parents of children and children
**Scientific value**			
Could increase target-specific information	6	[34–36, 73–75]	Children [36, 74, 75]
Could allow for better-quality data	2	[44, 46]	Newly diagnosed/disabled patients [44], patients from low- and middle-income countries [46]
Could increase scientific integrity	1	[21]	Women
Could be necessary to obtain the necessary data	1	[74]	Children
**Respect for persons**			
Could lead to upholding participants’ right to make autonomous decisions	3	[39, 46, 81]	Patients with Parkinson’s disease [39, 81], patients from low- and middle-income countries [46]
**Efficiency**			
Could accelerate the timelines of the study	1	[46]	Patients from low- and middle-income countries
Could reduce costs	1	[46]	Patients from low- and middle-income countries

### Non-maleficence

Most reasons that have been provided against including vulnerable populations in FIH research are based on non-maleficence. For instance, authors argue that certain patient groups such as children [15, 36] and pregnant women [34] could face a higher risk of harm from participating in FIH trials [15, 34, 36]. Whereas including patients with depression [43], patients at risk of dying by suicide [36], and patients with psychiatric diseases [36] could present ‘‘additional risks’’ of harm to participants [36, 43]. Authors noted that patients with unstable psychiatric diseases, lack of (adequate) caregivers, inadequate social support, or a history of poor compliance could not significantly benefit from the intervention [41]. Furthermore, within the context of deep brain stimulation, enrolling patients who suffer from extreme anxiety could be an obstacle for awake stereotactic surgery, negatively affecting their well-being [43]. Less advantaged patients could experience greater long-term costs [55] or lifestyle difficulties [55]. Additionally, one publication suggested that women could experience more (serious) adverse effects due to hormonal changes [11]. Further reasons were presented against including children in FIH research. For instance, authors noted that including children could lead to unnecessary treatment [15], as sometimes the child’s disease might not progress to an advanced stage. Another worry is that a child’s participation in FIH research might interfere with alternative treatment options that could become available to them in the future [15]. Lastly, authors noted that long-term follow-ups could place a greater burden on children than they would on adults [15].

### Beneficence

Despite the reasons against including vulnerable populations based on non-maleficence, authors have also highlighted reasons to include them based on the principle of beneficence. For instance, they noted that including vulnerable populations in FIH research could ensure that these groups derive greater benefits from future interventions [17, 34–36, 46, 73–75]. Further considerations highlighted the importance of including specific vulnerable populations. For instance, authors note that children could enjoy potential benefits from the intervention the longest [15, 74]. In some contexts, such as bio-artificial organ technologies, children could also suffer ‘‘…less damage to the body’’ (p.11) [15]. Participating in FIH research could furthermore prevent children from developing serious future complications [15]. Within the context of stem cell research for spinal cord injury (SCI) patients, FIH research could particularly benefit newly diagnosed or disabled individuals [76] whereas in the context of xenotransplantation, infants or children could gain the most benefits [36, 42, 70, 75]. Additionally, within the context of xenotransplantation, some authors argued that patients at risk of dying by suicide could be considered for inclusion in FIH research due to the positive effect the intervention might have on their quality of life [36, 72]. In the context of gene therapy, authors noted that in some cases younger children (under 12 years old) could specifically benefit from the intervention [58, 77]. Similarly, in the context of testicular tissue re-implementation, they noted that including prepubertal boys, who face highly-gonadotoxic therapies, are mostly likely to benefit from participating in FIH research [78]. Lastly, King and Cohen-Haguenauer that by including children in FIH gene therapy trials, children and their parents could derive psychological benefits [58].

### Scientific value

Authors have highlighted concerns regarding the scientific value of data obtained from vulnerable populations. For instance, some publications expressed that patients from low- or middle-income countries could have more undiagnosed diseases and untreated morbidities, which could affect the quality of data [30, 46]. Furthermore, it could be challenging to provide the necessary conditions to ensure high technical standards and safety of the research participants in low- and middle-income countries [30, 46]. In newly diagnosed or disabled patients it could be challenging to differentiate between spontaneous recovery and recovery attributed to the intervention [20, 22]. Authors also highlighted that within the context of xenotransplantation, including patients with unstable psychiatric diseases, lack of (adequate) caregivers, inadequate social support or a history of poor compliance could confound efforts to fairly evaluate the procedure’s safety and efficacy [41]. One publication asserted that including patients at risk of dying by suicide and those with psychiatric disorders could jeopardize the generalizability of knowledge obtained from the trial [36]. Finally, authors mentioned that elderly patients could have more co-morbidities, affecting the quality of data [46].

However, including vulnerable populations could also increase the scientific value of data. For instance, authors pointed out that incorporating vulnerable populations into FIH trials could yield target-specific insights about these groups [34–36, 73–75]. In the field of cancer nanomedicine, one argument supports including newly diagnosed patients in FIH research. Their participation could enhance data quality [44] because they have not undergone prior treatment and therefore have not developed resistance to cancer therapies yet. The same goes for patients from low- and middle-income countries, who are more likely to be treatment naïve [46]. In other contexts, such as paediatric liver organoid transplantation, obtaining essential data without including children could be impossible [74]. Lastly, one publication noted that including more women in FIH research could increase investigators’ scientific integrity by not skewing research protocols towards a norm of middle-aged men [21].

### Efficiency

Several reasons against including vulnerable populations were given based on trial efficiency. Authors suggested that it could be preferable to exclude certain vulnerable populations from FIH trials to minimize long-term setbacks in the research field [49]. Furthermore, within the context of deep brain stimulation, including patients who suffer from depression could influence pre- and postoperative measures [43]. Authors provided a final reason concerning patients from low-income countries; including these patients could introduce (more) cultural obstacles [30].

However, two reasons were provided for including patients from low- and middle-income countries in FIH trials based on efficiency. For some FIH trials, the pool of eligible patients is very small. Authors noted that in such cases, expanding trial recruitment to low- and middle-income countries can help accelerate the timelines of the study [46]. Furthermore, one publication noted that including patients from low- and middle-income countries can reduce costs, for instance, related to labour, recruitment and regulatory compliance [46] (Table 9).

### Respect for persons

Further considerations were made regarding respect for persons. Authors expressed concerns that various vulnerable populations could be affected in their capacity to provide informed consent [10, 15, 17, 20, 33–35, 39, 41, 46, 55, 59, 65, 74, 76, 79, 80]. This concern was particularly highlighted for patients with cognitive impairments [10, 33, 35, 39, 55, 79, 80], newly diagnosed or disabled individuals [10, 20, 41, 65, 76], children [15, 17, 34, 35, 46, 55, 59, 74], parents of dying infants [46] and patients with a mental disability [55]. In addition, newly diagnosed or disabled individuals could also be more susceptible to therapeutic misconception [10, 20, 49, 63, 64]. Lastly, authors noted that vulnerable populations could be more vulnerable to exploitation [4, 28, 30, 35, 49] or undue influence [49, 76] (Table 10).

One argument, to include patients with Parkinson’s disease who might experience mental capacity decline and patients from low- and middle-income countries, was given based on respect for persons. Authors asserted that these patients should retain the right to make autonomous decisions [39, 46, 81].

### Justice

One publication asserted that from the perspective of justice, it would be unfair to recruit vulnerable populations, due to their status as ‘‘being vulnerable’’ [10].

Nevertheless, most reasons for including vulnerable populations were given based on justice. Authors highlighted that including vulnerable populations in FIH research could enhance the equitable distribution of risks and benefits among prospective beneficiaries [73]. For children who have no alternative treatment options anymore, authors argued that it would be just if investigators included these patients and provided them with what could be their last potentially beneficial treatment option [6, 75]. In the context of paediatric liver organoid transplantation, including children in FIH research could furthermore provide patients and their families ‘‘a voice’’ [74]. Other reasons were given for including (more) patients from low-income countries. Authors argued that supporting FIH research in low-income countries through local participation could help alleviate the dependency of these countries on their wealthier counterparts [30], increase economic activity by encouraging research into more innovative products [30], facilitate healthcare infrastructure development [30] and drive capacity building for local ethical review [30].

## Should FIH trials be conducted with diverse participant groups?

Another discussion within the literature applies to the diversity of participant groups for FIH trials. While authors provided no specific reasons against including diverse participant groups, they did give reasons in favour of including diverse participant groups. For an overview of these reasons see Table 11.

**Table 11:** Reasons to include diverse participant groups (N = 7)

Narrow reason	N	Reference(s)
Justice		
Including diverse participant groups		
Could increase equitable distribution of risk and benefit in populations of prospective beneficiaries	7	[19, 21, 23, 34, 73, 80, 84]
Could decrease recruitment based on privilege	1	[10]
Could increase equal opportunity across eligible patient groups	1	[19]
Could be more just	1	[85]
Scientific value		
Could increase target-specific information which allows participants to benefit (more) from future interventions	8	[4, 11, 19, 46, 68, 73, 82–84]
Could enhance the value of the data by improving the generalizability	3	[4, 19, 30]
Could allow for assessing safety in different pathophysiological conditions	1	[83]

### Scientific value

From a scientific value perspective, including diverse participant groups could lead to more target-specific information for various participant subgroups [4, 11, 19, 46, 73, 82–84]. For instance, older HVs exhibit different pathophysiological characteristics than their middle-aged or younger counterparts [83], including participants from different ages in FIH research could therefore assess safety in different pathophysiological conditions. Finally, including a diverse group of participants could also increase the quality of the data by enhancing its generalizability [4, 19, 30].

### Justice

Most of the reasons that were given to include diverse participant groups were based on justice. Authors noted that including diverse participant groups could stimulate an equitable distribution of risks and benefits among prospective beneficiaries [19, 21, 23, 34, 68, 73, 80, 84]. Others emphasize that recruiting participants who mirror the target population(s) and encompass diversity in age, sex and ethnicity inherently adheres to the principle of justice [85]. Furthermore, recruiting diverse participant groups could decrease recruitment based on privilege [10] and stimulate equal opportunity across all eligible patient groups [19].

## Discussion

To our knowledge, this review offers the first comprehensive overview of reasons for and against the inclusion of potential participant groups in FIH trials. In this section, we offer some critical, overarching reflections on the results. We will conclude by presenting the review’s strengths and limitations.

### Vague or ambiguous reasoning

One issue that stood out during the data extraction process of this review was the number of reasons authors gave for or against including a participant group without any further explanation or justification. While we acknowledge that some reasons are self-evident and do not necessitate further elaboration, we believe that a significant portion of the reasons presented in this review remain unnecessarily unclear in their original publications. This lack of clarity can be attributed to reasons being either inherently vague, with the authors failing to provide adequate argumentation, or ambiguously formulated, allowing for multiple interpretations.

Interestingly, we found no correlation between vague or ambiguous reasoning and specific publication characteristics. For instance, there was no clear distinction between original research articles and reviews, nor between publications in medical versus ethical journals, or across different research fields regarding the presence or absence of vague and ambiguous reasoning. Thus, vague or ambiguous reasoning appears to be a widespread issue in the literature. For instance, authors argued that patients with MASD may not be the ideal participant group for medical reasons [29]. However, what (type of) medical reasons the authors are referring to remains unclear. Similarly, arguing against including patients in FIH research because it might increase difficulties in, and costs of, the recruitment process [4] does not explain what (type of) difficulties can be expected when recruiting patients instead of HVs. The same applies to the argument that including patients with depression or other psychiatric disorders, or people at risk of dying by suicide, could introduce ‘‘additional risks’’ [36, 43]; as the original texts do not detail what additional risks are being referred to. Examples of reasons that allow for multiple interpretations include the argument against including vulnerable populations due to their status as ‘being vulnerable’ [10], where the interpretation depends on the reader’s understanding of ‘vulnerable’ in this context. Another example is the argument for including children because it would provide them and their families with a voice [74], which can be interpreted in various ways depending on what ‘having a voice’ means in this context.

We believe that in ethical reasoning concerning the participant selection for FIH trials, it is crucial to present well-structured arguments. Failing to do so not only leaves readers unconvinced or confused but also obscures how the argument relates to underlying ethical values. Of particular concern are the ambiguous and vague reasons presented against including vulnerable populations. These poorly defined reasons, lacking adequate justification for the exclusion of vulnerable groups, may inadvertently contribute to their vulnerability by denying them access to contribute to and participate in research that would benefit their exact population [86]. Therefore, it is essential to offer well-structured arguments for and against including certain groups in FIH trials. This can be achieved, for instance, by offering a justification for the reasons one presents or by considering how readers might interpret the reasons. If reasons allow for multiple interpretations, authors should clarify the intended meaning behind them.

### No mention of the moral theory, framework, or method used

Not only do authors provide reasons without justifications, they also rarely mention or explain which moral theory, framework, or method underpins their arguments and conclusions. Most publications seem to draw on some form of Principlism or other mid-level moral theories to address the ethics of participation selection in FIH trials. Such approaches typically explore and identify important principles, values, or criteria that then dictate the morally appropriate course(s) of action. For instance, Kögel and colleagues developed a multicriteria approach to participant selection in FIH cardiac xenotransplantation trials, in which the criteria of (1) medical need, (2) capacity to benefit, (3) patient choice, and (4) compliance (as an exclusion criterion) dictate which patients should be included [42]. In contrast, a more bottom-up approach is used by Bretzner and colleagues, who compare three different groups of patients with SCI to determine which distinctive features amongst these groups make their inclusion in a FIH trial of stem cell therapy for SCI more or less morally desirable [49].

However, even in these examples where the moral framework or method used is more apparent to the reader – authors do not explicitly acknowledge or mention it. This lack of clarity is problematic because it hinders readers from fully understanding and critically evaluating the foundation of the authors’ conclusions, specifically the moral reasoning or theoretical commitments guiding their analysis. Clearly stating the moral framework or method not only clarifies how the conclusions were reached but also allows readers to assess whether the conclusions logically follow from the stated premises. Moreover, such transparency enhances the quality and interpretability of the bioethical debate on this topic and encourages constructive discussion. Therefore, we recommend that authors writing on the ethics of participant selection in FIH trials explicitly state which moral theory, framework, or method they use to guide their analysis.

### Beneficence as an important theme for including potential participant groups in FIH trials

Interestingly, authors provided reasons based on beneficence for excluding as well as including potential participant groups. Reasons against inclusion were grounded in the idea that trial participation would not provide benefits to certain (sub)groups, whereas reasons for inclusion centred on the possibility that FIH trial participation could offer (in)direct benefits to participants. For instance, in the context of HVs, authors argued that for individuals at risk of developing the targeted disease, participating in a FIH could delay or prevent the onset of disease, thereby improving overall health outcomes [15]. Similarly, they argued that including special forces personnel could receive direct benefits by optimizing their performance in high-risk missions [37]. With regards to patients, authors argued that including patients with LASD could allow them access to a superior means of treating their disease [26], whereas others argued for patients with MASD because it could treat some of their debilitating symptoms [43] or lead to more effective treatment [56]. While it is unsurprising that beneficence emerged as an important theme in participant selection for FIH trials generally, its use as a justification for *including* potential participant groups remains contested.

Importantly, the goal of FIH research is primarily to determine the safe dose range for further clinical development [1]. FIH trials typically involve administering drug candidates or therapies to participants in low, subtherapeutic doses to determine the safe dose range [9, 12]. Typically, any assessment of the potential therapeutic effectiveness of these drug candidates occurs later in the clinical development process. As a result, the potential of FIH trials to benefit participants is uncertain. While some explicitly argue that FIH trials are not designed to benefit participants [6], others acknowledge that researchers can, and often do, incorporate trial design elements – such as accelerated dose escalation or specific inclusion strategies – to increase the likelihood of providing benefits to participants, but that therapeutic claims cannot be made based on intent alone [87]. Furthermore, there is little scientific evidence that participation offers any therapeutic benefit to participants, as most FIH trials neither demonstrate safety nor efficacy [87, 88]. Therefore, as Jonathan Kimmelman argues, claims of therapeutic benefit in FIH trials should be met with scepticism [88].^8^ Yet, the results of this review indicate that assumptions about benefits to trial participants are highly prevalent in the literature. The debate on whether FIH trials actually provide such benefits raises questions about the extent to which beneficence to participants should justify including potential participant groups.

### Difficulties in identifying and defining vulnerable populations

Finally, something we noticed and experienced while writing this review is the difficulty of identifying and defining vulnerable populations. Some authors explicitly used the term ‘vulnerable populations’. Although nearly all of those authors briefly defined which subgroups they considered as vulnerable populations, one publication used the term ‘vulnerable populations’ without further elaboration [10]. Among the publications that did list examples of vulnerable populations, nearly all mentioned children and women of childbearing age, but the number of groups listed varied significantly among the publications. It is important to note that authors cannot be blamed entirely for providing no or inconsistent definitions of vulnerable populations, as this remains a topic of ongoing debate in the literature.

The philosophical literature broadly defines three conceptions of vulnerability: inherent vulnerability, categorical vulnerability, and contextual vulnerability [89, 90].^9^ Inherent vulnerability relates to vulnerabilities that are intrinsic to the human experience; they arise from our corporeality [90]. All people are for instance inherently vulnerable to hunger, sleep deprivation or social isolation. The second conception of vulnerability is categorical vulnerability. This approach labels members of groups vulnerable when they share a salient feature that might make them vulnerable [91, 92]. For instance, children may be considered a vulnerable population because they all share the feature of not having fully developed autonomous decision-making capacities. Finally, there is the contextual notion of vulnerability, which identifies groups as vulnerable under specific circumstances or situations [90, 91, 93]. For instance, HVs with low incomes or patients from low-income countries may be more vulnerable to exploitation when monetary incentives are involved [4, 28, 41].

Based on these three approaches to vulnerability, we decided to categorize all (sub)groups that are vulnerable in one or more of these three ways as vulnerable populations.^10^,^11^ Nevertheless, researchers must consider the potential implications of adopting a categorical approach to vulnerability. Some of the reasons provided by authors in this review rely on this categorical perspective, as exemplified by the argument against including women in FIH research due to their potential for more (serious) adverse effects [11]. While there may be instances where categorical vulnerability-based reasons are justified, for instance, when it concerns children, we should generally avoid automatically excluding participant groups based on a salient feature they share [93]. Default exclusion undermines their long-term interests and leaves these groups vulnerable to harm, due to a lack of scientific evidence on the safety and/or efficacy of future interventions in these groups [94, 95]. Instead, our focus should shift towards implementing safeguards to protect participants from contextual vulnerability, for instance, by thinking about ways in which their contextual vulnerability may be lessened. Based on such an approach, if including HVs with low incomes and patients from low-income countries could be vulnerable to exploitation [4, 28, 30, 35], this should not, by definition, lead to their exclusion from FIH research. Instead, we should think about ways to mitigate this contextual vulnerability for these groups, for instance, by not offering participants monetary compensation for their participation in FIH trials or spending more careful attention to the informed consent process. Future research must be conducted on how to identify and define vulnerable populations in FIH research. Key questions to address include: Do vulnerable populations exhibit varying degrees of vulnerability, and if so, how should these differences be managed? What are the most effective strategies for mitigating contextual vulnerabilities amongst potential participant groups, and how can we ensure that additional safeguards are feasible and effective in clinical practice?

### Strengths and limitations

This review has several strengths and limitations. Firstly, to our knowledge, this review represents the first comprehensive overview of the reasons for and against including potential participant groups in FIH trials. As previously mentioned, we categorized all subgroups into six overarching categories, which creates a clear structure. At the same time, this also means that the results may lack some precision since the complexity between and nuances within subgroups cannot be fully captured within broad categories. Additionally, interpretation plays an inevitable role in classifying reasons across participant categories and mapping broad themes to narrow reasons. Correspondingly, different research teams may have made different choices in data coding and categorization. Furthermore, our review has focused on FIH trials. Although all FIH trials, including phase 0 trials, fall under the umbrella of phase 1 trials, not all phase 1 trials are FIH trials. Notably, there is also a significant body of literature discussing the ethics of phase 1 trials, some of which may have been written to apply (also) to FIH trials, but which was not included in this review to allow for a comprehensive analysis of the reasons within this specific subset of early-phase clinical research. Lastly, the results of this review are meant to be interpreted as descriptive rather than normative. We have not provided a systematic quality assessment of the included reasons, nor does such an instrument exist (yet) for normative literature [96]. Hence, we leave it up to the reader to evaluate and, if applicable, assign normative weight to the reasons outlined in this review. The results presented in this review can serve as the foundation for such evaluations.

## Conclusions

FIH trials represent the first stage of clinical research, with novel therapies being tested in humans for the first time. Determining which potential participant groups should be allowed to take part in these trials poses a significant challenge for researchers, clinicians, sponsors, and ethicists. This systematic review provides the first comprehensive overview of the reasons presented for and against the inclusion of potential participant groups in FIH research. 181 reasons were identified across six potential participant categories. Our findings indicate that the reasons provided by authors in the existing literature on this subject sometimes remain unnecessarily ambiguous or lack sufficient argumentation, and most of the time, authors do not state which moral theory, framework, or method they use to guide their analysis. Furthermore, beneficence emerged as an important theme for including potential participant groups, even though the possibility of FIH trials providing benefits to participants is contested, raising questions about its use as a justification for including participants in FIH trials. Lastly, identifying and defining vulnerable populations remains an ongoing challenge in the literature, emphasizing the need for further research and analysis. The results of this review can provide those who are dealing with specific questions regarding the participant selection for FIH trials with an overview of considerations that could be relevant to critically reflect upon, and offer guidance for further normative inquiry.

## Supplementary Information

Additional file 1

Additional file 2

## Data Availability

All data generated and analyzed during this study are included in this published article and were publicly available at the time of submission.

## Abbreviations

FIH: First-in-human
HVs: Healthy volunteers
Patients with LASD: Patient with less advanced-stage disease
Patients with MASD: Patients with more advanced-stage disease
SAD: Single ascending dose
PK: Pharmacokinetics
PD: Pharmacodynamics
SCI: Spinal cord injury

## Footnotes

1 Notably, the reasons and underlying ethical values mentioned most frequently in the existing literature do not necessarily carry the most normative weight.

2 The publications included in the review use the label ‘FIH’ to refer to trials in which an intervention is being tested in humans for the first time. However, there is no consensus on the exact definition of ‘first-time testing in humans’. For instance, it is unclear whether interventions that have never been tested in humans before, but are similar to already existing treatments (such as ‘’me-too’’ drugs) should be classified as FIH. Similarly, ambiguity exists for interventions that have been tested in humans before but are being tested for the first time with a new route of administration. Therefore, further work is needed to develop a clearer conceptual framework for FIH.

3 Given the ongoing debate on including HVs or patients (in general) in FIH trials, we have chosen to incorporate a general patient category alongside the other two patient categories.

4 Searches for the second update (09-01-2025) were conducted with different starting dates due to database restrictions. For some databases, publications were retrieved since the last search on 14-05-2024, while others allowed searches only within the past year (from 09-01-2024 to 09-01-2025). To address potential overlaps, a thorough de-duplication process was implemented.

5 Non-maleficence is an ethical value which emphasizes that causing intentional or unnecessary harm through one’s actions should be avoided.

6 Where animal pathogens are transmitted to the xenograft recipient and can then be transmitted from human to human.

7 Notably, many reasons were also provided against including specific subgroups of patients. However, these are mostly discussed within the section on vulnerable populations.

8 Notable, some FIH trials are part of a combined FIH/Phase 2 trial or trials involving expansion cohorts, extending its scope beyond initial safety assessment.

9 These classifications of vulnerability into builds on broader ethical discussions about vulnerability, such as Robert Goodin’s account of moral responsibility toward those unable to protect themselves.

10 Some publications explicitly highlighted seriously ill patients as a vulnerable population. However, for the purpose of this review, we have decided to categorize this patient group under the category of patients with MASD, since the reasons for this group were in line with those of patients with MASD.

11 It should furthermore be noted that all participants in FIH research—as are all human beings—are vulnerable to some extent. However, to do justice to the discussion within the literature around the in- and exclusion of vulnerable populations we decided to create a separate category for populations which may be more vulnerable than others.
