# Supplementary material for: Who should be included in first-in-human trials? A systematic review of reasons
Source: J Transl Med. 2025 Jun 11;23:649. doi: 10.1186/s12967-025-06550-y (PMC12160108; doi:10.1186/s12967-025-06550-y)
Supplement: Supplementary file 1 — Additional file 1 [file 12967_2025_6550_MOESM1_ESM.docx]

*Additional file 1. The search string*

| **Search string 1 (PubMed, Embase, The Philosopher’s Index, Web of Science, Academic Search Premier)s** | ((‘’First in human’’ OR ‘’First-in-human’’ OR ‘’FIH trial’’ OR ‘’FIH study’’ OR ‘’FIH studies’’ OR ‘’FIH research’’) AND (‘’Patient selection’’ OR ‘’Healthy volunteers’’ OR Participants OR Patients OR Subjects OR Volunteers) AND (‘’Decision Making’’ OR Philosophy OR Ethics OR Ethical OR Justify OR Justified OR Justifying Or Justifiable OR Justification OR Reason OR Reasons OR Reasoning OR Argument OR Arguments OR Argumentation OR Criteria OR Ground OR Grounds OR Motivation OR Motivations OR Explanation OR Explanations OR Drive OR Drives)) |
| --- | --- |
| **Search string 2 (PhilPapers, Leiden University Library)** | First-in-human |
