# Supplementary material for: Who should be included in first-in-human trials? A systematic review of reasons
Source: J Transl Med. 2025 Jun 11;23:649. doi: 10.1186/s12967-025-06550-y (PMC12160108; doi:10.1186/s12967-025-06550-y)
Supplement: Supplementary file 2 — Additional file 2 [file 12967_2025_6550_MOESM2_ESM.docx]

*Additional file 2. Publications characteristics*

| Publication | Publication Type | Field of journal | Research field the publication focusses on | The extent to which the topic is addressed |
| --- | --- | --- | --- | --- |
| Ahmed et al., (2020)(14) | Review | Medicine | Oncology drug development | Extensively |
| Baker (2016)(64) | Review | Methodology | Tissue engineering | Briefly |
| Barker et al., (2015)(62) | Perspective | Medicine | Stem cell research | Briefly |
| Barket & de Beaufort (2013)(33) | Review | Medicine | Stem cell research | Briefly |
| Bobier (2024)(23) | Research article | Medical ethics | Clinical trials (general) | Substantially |
| Bompart (2019)(24) | Research article | Medical ethics | Clinical trials (general) | Extensively |
| Bretzner er al., (2011)(49) | Perspective | Medicine | Stem cell research | Extensively |
| Bunnik et al., (2022)(38) | Report | Medicine | Bio-artificial organ technologies | Briefly |
| Burt et al., (2022)(73) | Review | Medicine | Clinical trials (general) | Briefly |
| Chapman & Scala (2012)(65) | Research article | Ethics | Embryonic stem cell research | Briefly |
| Chapman (2011)(4) | Research article | Bioethics | Clinical trials (general) | Substantially |
| Coates et al., (2020)(51) | Research article | Medicine | Neuromodulation | Briefly |
| Collins (2007) (27) | Book chapter | Pharmacology | Clinical trials (general) | Briefly |
| Cote et al., (2017)(66) | Editorial | Medicine | Stem cell research | Briefly |
| Daverio (2019)(34) | Research article | Law | Clinical trials (general) | Substantially |
| Dawson et al., (2003)(29) | Report | Medicine | Cell research | Briefly |
| de Jongh et al., (2022)(15) | Review | Medicine | Bio-artificial organ technologies | Substantially |
| de Las Heras et al., (2022)(9) | Research article | Medicine | Oncology drug development | Substantially |
| de Melo Martín et al., (2018)(79) | Perspective | Medicine | Stem cell research | Briefly |
| Devos et al., (2022)(43) | Review | Medicine | Deep brain stimulation | Substantially |
| Dresser (2009)(5) | Research article | Medical ethics | Stem cell research | Substantially |
| Dresser (2017)(16) | Research article | Medical ethics | HIV-remission research | Briefly |
| Dubé et al., (2022)(85) | Research article | Medical ethics | HIV remission research | Briefly |
| Fung & Kerridge (2013)(39) | Research article | Bioethics | Stem cell research | Briefly |
| Gibson (2024)(71) | Commentary | Medical ethics | Xenotransplantation | Extensively |
| Gilbert et al., (2012)(52) | Research article | Bioethics | Optogenetic research | Briefly |
| Gilbert et al., (2014)(53) | Research article | Bioethics | Optogenetic research | Briefly |
| Greene & Master (2018)(37) | Perspective | Bioethics | Military enhancement | Briefly |
| Gupta (2020)(80) | Book chapter | Medicine | Neuroscience | Briefly |
| Gusmano (2022)(68) | Commentary | Bioethics | Xenotransplantation | Briefly |
| Habets et al., (2016)(54) | Research article | Bioethics | Stem cell research | Briefly |
| Habets et al., (2016)(63) | Commentary | Medicine | Stem cell research | Briefly |
| Habets et al., (2017)(6) | Perspective | Pharmacology | Clinical trials (general) | Briefly |
| Harris & Gilbert (2022)(17) | Book chapter | Military and humanitarian health ethics | Military medicine research | Substantially |
| Harris et al., (2022)(55) | Research article | Medicine | Stem cell research | Briefly |
| Hendriks et al., (2022)(25) | Research article | Bioethics | Embryonic research | Briefly |
| Hewson et al., (2013)(76) | Expert review | Medicine | Stem cell research | Briefly |
| Hey & Kimmelman (2014)(45) | Research article | Ethics | Clinical trials (general) | Briefly |
| Hug & Hermerén (2012)(18) | Research article | Medical ethics | Stem cell research | Extensively |
| Hug (2020)(69) | Perspective | Medicine | Cell research | Briefly |
| Hurst et al., (2015)(10) | Target article | Bioethics | Stem cell research | Substantially |
| Hurst et al., (2022)(36) | Research article | Medicine | Xenotransplantation | Extensively |
| Hurst et al., (2024)(75) | Commentary | Medical ethics | Xenotransplantation | Extensively |
| Iersel et al., (2022)(13) | Perspective | Pharmacology | Clinical trials (general) | Substantially |
| Iyer et al., (2021)(19) | Review | Medicine | Paediatric gene therapy | Substantially |
| Ji et al., (2023)(32) | Review | Pharmacology | Oncology drug development | Briefly |
| Kapirit et al., (2011)(30) | Perspective | Public health | Clinical trials (general) | Extensively |
| Karakunnel et al., (2018)(11) | Review | Pharmacology | Drug development | Substantially |
| Kimmelman & London (2011)(48) | Perspective | Medicine | Clinical trials (general) | Briefly |
| Kimmelman (2007)(26) | Commentary | Medicine | Gene therapy | Extensively |
| Kimmelman (2009)(46) | Book | Bioethics | Gene therapy | Substantially |
| Kimmelman (2024) (47) | Book chapter | Medicine | Gene- and cell therapy | Briefly |
| King & Cohen-Haguenauer (2008)(58) | Research article | Medicine | Gene therapy | Substantially |
| King & Perrin (2014)(20) | Review | Medicine | Stem cell research | Briefly |
| King (2014)(22) | Book chapter | Medicine | Regenerative medicine | Briefly |
| Kögel et al., (2024)(42) | Research article | Medical ethics | Xenotransplantation | Extensively |
| Koonrungsesomboon et al., (2016)(21) | Review | Medicine | Clinical trials (general) | Substantially |
| Kurihara (2011)(82) | Review | Pharmacology | Clinical trials (general) | Briefly |
| Laspro et al., (2024)(60) | Research article | Bioethics | Whole eye transplantation | Substantially |
| Leach et al., (2021)(12) | Report | Pharmacology | Clinical trials (general) | Briefly |
| London et al., (2010)(67) | Research article | Medicine | Innovative therapies | Briefly |
| Mitchell & Ives (2024)(78) | Editorial | Medicine | Human reproduction research | Briefly |
| Mundy et al., (2023) (61) | Review | Pharmacology | Drug development | Substantially |
| Niemansburg et al., (2013)(40) | Review | Medicine | Orthopaedic research | Briefly |
| Niemansburg et al., (2014)(56) | Research article | Medicine | Cardiovascular medicine | Substantially |
| Park et al., (2013)(83) | Research article | Medicine | Clinical trials (general) | Extensively |
| Petrini (2010)(35) | Research article | Interdisciplinary | Clinical trials (general) | Substantially |
| Petrini (2011)(28) | Review | Medicine | Clinical trials (general) | Briefly |
| Pierson et al., (2022)(41) | Research article | Medicine | Xenotransplantation | Extensively |
| Reese et al., (2023)(72) | Editorial | Medicine | Xenotransplantation | Briefly |
| Reichart et al., (2023)(70) | Review | Medicine | Xenotransplantation | Briefly |
| Rood & Cooper (2006)(59) | Perspective | Medicine | Xenotransplantation | Briefly |
| Satalkar et al., (2016)(44) | Research article | Medicine | Cancer nanomedicine | Substantially |
| Schneemann et al., (2020)(74) | Review | Medicine | Pediatric liver organoid transplantation | Substantially |
| Siobhan et al., (2020)(77) | Book chapter | Medicine | Gene therapy | Briefly |
| Solbakk & Zoloth (2011)(81) | Commentary | Medicine | Human embryonic stem cell research | Briefly |
| Sugarman (2008)(57) | Review | Medicine | Embryonic stem cell research | Briefly |
| Tranter et al., (2013)(31) | Review | Pharmacology | Monoclonal antibody research | Briefly |
| van Kempen et al., (2024)(84) | Commentary | Medical ethics | Xenotransplantation | Extensively |
| Wirth et al., (2011)(50) | Commentary | Medicine | Clinical trials (general) | Extensively |
